# Real‐world efficacy of atezolizumab in non‐small cell lung cancer: A multicenter cohort study focused on performance status and retreatment after failure of anti‐PD‐1 antibody

**DOI:** 10.1111/1759-7714.13824

**Published:** 2021-01-15

**Authors:** Naoki Furuya, Makoto Nishino, Kazushige Wakuda, Satoshi Ikeda, Takashi Sato, Ryota Ushio, Shigeru Tanzawa, Masafumi Sata, Kentaro Ito

**Affiliations:** ^1^ Division of Respiratory Medicine, Department of Internal Medicine St. Marianna University School of Medicine Kawasaki Japan; ^2^ Department of Experimental Therapeutics National Cancer Center Hospital Tokyo Japan; ^3^ Division of Pulmonary Medicine, Department of Medicine Keiyu Hospital Yokohama Japan; ^4^ Division of Thoracic Oncology Shizuoka Cancer Center Shizuoka Japan; ^5^ Department of Respiratory Medicine Kanagawa Cardiovascular and Respiratory Center Yokohama Japan; ^6^ Respiratory Disease Center, Yokohama City University Medical Center Yokohama Japan; ^7^ Division of Medical Oncology, Department of Internal Medicine Teikyo University School of Medicine Tokyo Japan; ^8^ Division of Pulmonary Medicine, Department of Medicine Jichi Medical University Tochigi Japan; ^9^ Respiratory Center, Matsusaka Municipal Hospital Matsusaka Japan

**Keywords:** anti‐PD‐L1, atezolizumab, non‐small cell lung cancer, retreatment

## Abstract

**Background:**

Atezolizumab is a programmed death‐ligand 1 (PD‐L1) targeted monoclonal antibody that inhibits PD‐L1 interacting with its receptors PD‐1 and B7‐1, thereby enhancing anticancer immunity. Some real‐world efficacy and safety studies of anti‐PD‐1 antibody have been previously reported. However, there have been no reports investigating the efficacy of atezolizumab monotherapy in clinical practice which have focused on performance status and previous anti‐PD‐1 antibody treatment.

**Methods:**

We retrospectively reviewed consecutive advanced NSCLC patients who received atezolizumab monotherapy between April 2018 and February 2019 at eight institutions. A total of 152 patients with NSCLC were enrolled in this study.

**Results:**

A total of 38 patients (25%) had already been treated with anti‐PD‐1 treatment (nivolumab or pembrolizumab) before atezolizumab. The median OS and TTF was 384 days (12.8 months) (95% confidence interval [CI]: 206–424), and 42 days (1.4 months) (95% CI: 27–56) in all patients, respectively.

ECOG PS 0 had significantly longer OS (median OS; not reached, *p* < 0.0001) and TTF (median TTF; 63 days, *p* = 0.012) compared with PS 1 or 2–3. Most retreated patients were unable to continue atezolizumab for a longer period, but seven patients (18.4%) were able to continue atezolizumab over four months as an ICI retreatment.

**Conclusions:**

In previously treated advanced NSCLC patients, atezolizumab monotherapy demonstrated good efficacy and safety regardless of heavily treated patients in real‐world clinical practice, and ECOG PS 0 was a favorable predictive factor. The efficacy of retreatment with atezolizumab was limited but was well tolerated in patients treated with prior anti‐PD‐1 antibody.

## Graphical abstract

Swimmers plot of atezolizumab according to the efficacy of prior anti‐PD‐1 treatment. Seven patients (18.4%, 7/38) continued atezolizumab over four months as an ICI retreatment.

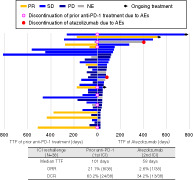

## INTRODUCTION

Atezolizumab is a programmed death‐ligand 1 (PD‐L1) targeted monoclonal antibody that inhibits PD‐L1 interacting with its receptors PD‐1 and B7‐1, thereby enhancing anticancer immunity.^1^

In the OAK trial, atezolizumab showed superiority for overall survival (OS) when comparing docetaxel as a second‐line treatment in patients previously treated with platinum doublet chemotherapy for non‐small cell lung cancer (NSCLC), regardless of PD‐L1 expression.^2^ Therefore, atezolizumab monotherapy became the standard care for advanced NSCLC for second‐line treatment. Subsequently, immune checkpoint inhibitor (ICI) monotherapy or combination therapy of ICI plus platinum doublet were investigated as first‐line treatment in randomized phase III trials.^3^, ^4^, ^5^, ^6^, ^7^ These phase III trials met the primary endpoint, and currently pembrolizumab monotherapy or combination therapy of ICI plus platinum doublet have become the standard treatment in patients with advanced NSCLC for first‐line treatment.

In Japan, atezolizumab was approved for the treatment of patients with advanced NSCLC in January 2018. However, since December 2015, anti‐PD‐1 antibodies (nivolumab and pembrolizumab) were already being used as the standard second‐line treatment in clinical practice for patients with previously treated advanced NSCLC. Some real‐world efficacy and safety studies have subsequently been reported.^8^, ^9^, ^10^ However, there have been no reports which have investigated the efficacy of atezolizumab monotherapy in clinical practice focusing on performance status and retreatment after failure of anti‐PD‐1 antibody treatment. Therefore, to evaluate this, we performed this retrospective study.

## METHODS

### Patients

We retrospectively reviewed consecutive advanced NSCLC patients who received atezolizumab monotherapy between April 2018 and February 2019 at eight institutions. A total of 152 patients with NSCLC were enrolled in this study. Clinical data were obtained from medical records according to study protocol. The clinical features included age, sex, Eastern Cooperative Oncology Group performance status (ECOG PS), smoking history, histology, *EGFR* mutation status, clinical stage (UICC eighth edition), number of prior regimens, prior ICI therapies, metastatic sites and PD‐L1 expression status before atezolizumab therapy. This study was approved by the institutional review board of each institution. PD‐L1 expression was evaluated by immunohistochemical stain (PD‐L1 IHC 22C3, pharmDx, Dako/Agilent, USA). Atezolizumab 1200 mg/bodyweight was intravenously administered every three weeks until disease progression or unacceptable toxicity.

### Outcomes and efficacy evaluations

The objectives of this study were to evaluate the efficacy and safety of atezolizumab in patients with advanced NSCLC with endpoints including overall survival (OS), time to treatment failure (TTF) with a data cutoff of August 2020. OS was determined as an interval from the first day of atezolizumab to any cause of death. TTF was defined as an interval from the first day of atezolizumab to the discontinuation of treatment for any reason. Tumor response was evaluated by the investigators according to the Response Evaluation Criteria in Solid Tumors (RECIST) version 1.1. The objective response rate (ORR) was defined as the proportion of patients with the best overall response for complete response (CR) or partial response (PR). Disease control rate (DCR) was defined as the proportion of patients with the best overall response for CR, PR, or stable disease (SD). Adverse events were graded according to the National Cancer Institute Common Terminology Criteria for Adverse Events (CTCAE) version 5.0.

### Statistical analysis

Survival curves were calculated by Kaplan–Meier method and compared by log‐rank test according to ECOG PS and PD‐L1 status. Statistical analyses were performed using Student's *t*‐test, χ^2^ tests and Fisher's exact test for continuous and categorical variables, respectively. Statistical analyses were performed using SPSS software, version 23.0 (SPSS Inc., Chicago, USA). Statistical significance was indicated by *p*‐values of less than 0.05.

## RESULTS

### Patient characteristics

A total of 152 patients were enrolled in this study for efficacy and safety analyses. The characteristics of all patients are listed in Table 1. ECOG PS 1 and adenocarcinoma were dominant characteristics in this study. In this cohort, 47 (30.9%) *EGFR* mutation positive patients were enrolled. A total of 38 patients (25%) had already been treated with anti‐PD‐1 treatment (nivolumab or pembrolizumab) before atezolizumab.

**TABLE 1:** Baseline characteristics of patients

	*N* = 152
Age, median (range)	71 (43–93)
Sex (male/female)	102/50
ECOG PS (0/1/2/3/unknown)	52/71/7/1/21
Smoking history (never/former/current/unknown)	36/89/19/8
Histology (a denocarcinoma/squamous/others)	122/20/10
Driver mutation (*EGFR*/*ALK*/none)	47 (30.9%)/3 (2%)/102 (67.1%)
Stage (III/IV/postoperative recurrence)	23/108/21
Number of prior regimens, median (range)	2 (2–13)
1/2/3/≥4	65/30/14/43
Prior treatment of immunotherapy (−/+)	114 (75%)/38 (25%)
Nivolumab	29
Pembrolizumab	8
Nivolumab and pembrolizumab	1
Metastatic site	
Brain (−/+/unknown)	96/39/17
Liver (−/+/unknown)	105/45/2
PD‐L1 expression (22C3)	
None (1% < TPS)	88 (57.9%)
Low (TPS: 1%–49%)	25 (16.4%)
High (TPS ≥ 50%)	14 (9.2%)
Unknown	25 (16.4%)

### Efficacy analyses

**FIGURE 1:**
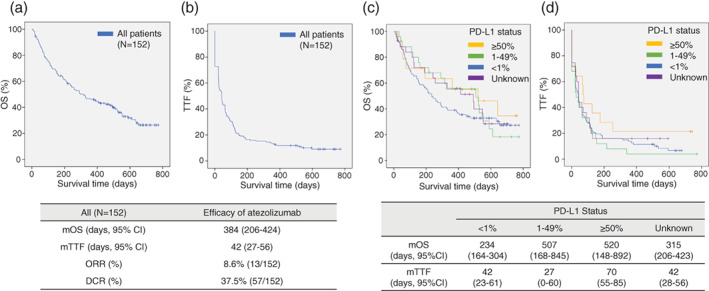
Kaplan–Meier survival curves of OS and TTF (a, b) in all patients, and according to PD‐L1 status (c, d)

At data cutoff (August 2020), 104 patients (68.4%) had died, and 14 patients (9.2%) received continuous atezolizumab treatment. The median follow‐up was 292 days (9.7 months). The median number of cycles of atezolizumab was three. The median OS and TTF was 384 days (12.8 months) (95% CI: 206–424), 42 days (1.4 months) (95% CI: 27–56) in all patients, respectively (Figure 1(a), (b)). According to PD‐L1 expression, in patients with PD‐L1 high (≧50%), median OS and TTF were longer compared with PD‐L1 low or none, but there were no significant differences. OS survival curves according to PD‐L1 expression nearly overlapped, especially for PD‐L1 ≥ 50% and PD‐L1 1%–49% (Figure 1(c)). In TTF survival curves, PD‐L1 ≥ 50% group demonstrated longer TTF (Figure 1(d)). All patients were assessed for therapeutic response. However, among the 152 enrolled patients, 14 were assessed as NE (not evaluated) as these patients were not scanned with computed tomography (CT) in clinical practice. ORR and DCR in all patients was 8.6% and 37.5%, respectively.

**FIGURE 2:**
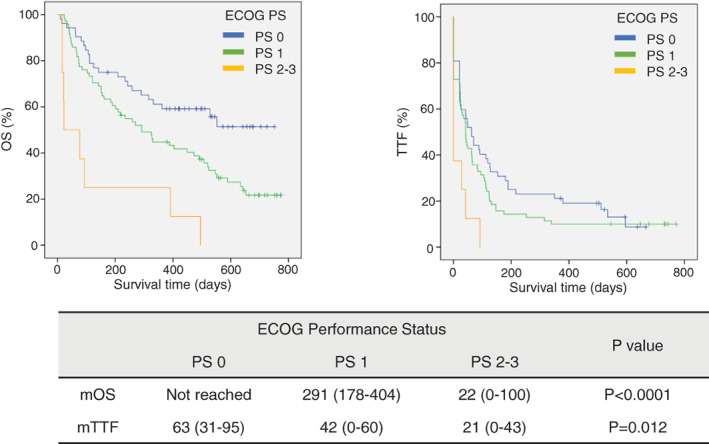
Kaplan–Meier survival curves of OS (a) and TTF (b) according to ECOG PS

Additional analyses according to ECOG PS, OS and TTF survival curves were clearly separated. ECOG PS 0 had significantly longer OS (median OS; not reached, *p* < 0.0001) and TTF (median TTF; 63 days, *p* = 0.012) compared with PS 1 or 2–3 (Figure 2(a), (b)).

**FIGURE 3:**
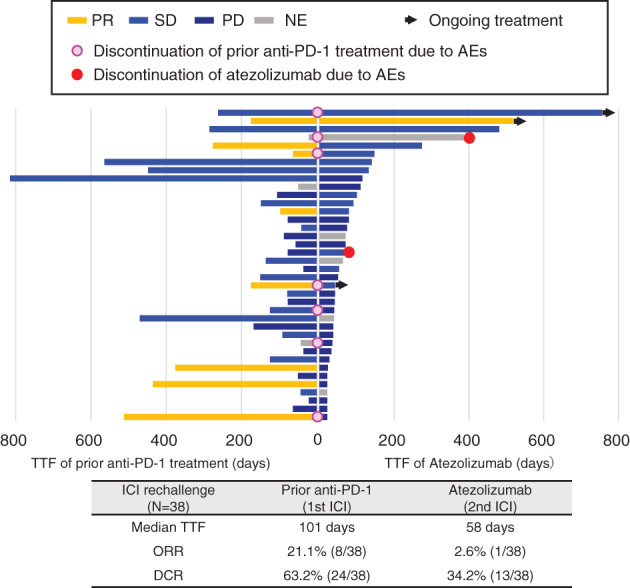
Swimmers plot of atezolizumab according to the efficacy of prior anti‐PD‐1 treatment (*N* = 38)

TTF, ORR and DCR of atezolizumab are shown according to the efficacy of prior anti‐PD‐1 treatment (*N* = 38, Figure 3). The median TTF was 56 days (2 months) in ICI retreatment subset. ORR and DCR was 2.6% (1/38) and 34.2% (13/38), respectively.

### Safety analyses

Five patients (3.3%) discontinued atezolizumab due to treatment related adverse events (AEs) which included three patients with interstitial pneumonia, one with liver dysfunction, and one with bronchial bleeding. However, there was no treatment‐related death. A total of 55 patients (36.9%) developed immune‐related adverse events (irAEs). Key safety data (irAE) are shown in Table 2. According to Figure 3, one patient developed an irAE during both prior ICI and atezolizumab. In this patient, the first irAE was hepatitis, while the second irAE was cholangitis.

**TABLE 2:** Immune‐related adverse events (irAEs) of any grade

	All patients (*N* = 152)	Prior anti‐PD‐1 (+) subset (*N* = 38)
irAE	36.9% (56)	23.7% (9)
Interstitial pneumonia	10.5% (16)	7.9% (3)
Skin rash	7.2% (11)	10.5% (4)
Liver dysfunction	17.1% (26)	7.9% (3)
Colitis	3.3% (5)	0% (0)
Thyroid dysfunction	6.6% (10)	2.6% (1)
Cholangitis	0.7% (1)	2.6% (1)

## DISCUSSION

To the best of our knowledge, this is the first and largest multicenter retrospective cohort study to investigate real‐world efficacy and safety of atezolizumab monotherapy in patients with advanced NSCLC. In the present study, ECOG PS was identified as a significantly favorable prognostic factor for OS and TTF. Some previous studies had already demonstrated the same results, but these studies enrolled only ICI naïve patients.^8^, ^9^, ^10^ In contrast, this study included patients that had been treated with anti‐PD‐1 treatment (nivolumab or pembrolizumab) before atezolizumab in 25% of this cohort. However, OS was not so poor despite relatively shorter TTF and lower ORR compared with a previous pivotal study (OAK trial).^2^ One reason for this was the post progression survival. At the data cutoff (August 2020), 138 patients had already discontinued atezolizumab. However, 118 patients (85.5%, 118/138) received subsequent chemotherapy after atezolizumab. A recent study (WJOG10217L) demonstrated higher ORR of chemotherapy after PD‐1 inhibitor treatment (CAP) compared with chemotherapy alone. OS of CAP in patients was 10.4 months.^11^ In our study, OS (12.8 months) was compatible with WJOG10217L.

The safety of atezolizumab was similar to previous studies. However, liver dysfunction (elevation of AST/ALT) was relatively high in frequency compared to previous prospective pivotal studies. One reason for this might be the enrollment of patients that were heavily treated with other regimens. In Japanese subsets, it is generally well known that interstitial pneumonia is found in higher frequency compared with other races. Recently we reported that honeycomb lung on high resolution CT (HRCT) was a risk factor of ICI‐induced pneumonitis.^12^ In the present study, three patients developed interstitial pneumonia, but honeycomb lung was not seen in these three patients. In clinical practice, HRCT should be carefully assessed at baseline.

In Japan, atezolizumab monotherapy was approved as a second‐line treatment for advanced NSCLC in January 2018. However, nivolumab and pembrolizumab had already been established as a standard second‐line treatment at that time. Therefore, some patients were treated with atezolizumab after nivolumab or pembrolizumab in clinical practice. A small case series previously investigated the efficacy of ICI retreatment.^13^ One clinical question is the efficacy of anti‐PD‐L1 antibody in patients who develop acquired resistance. To elucidate this, in Figure 3 additional analyses revealed no correlation between the efficacy of prior anti‐PD‐1 treatment and the efficacy of retreatment with atezolizumab. Most patients who had been retreated were unable to continue atezolizumab for a longer period, but seven patients (18.4%, 7/38) were able to continue atezolizumab over four months as an ICI retreatment. Retreatment with atezolizumab was also shown to be well tolerated (Table 2).

This study has several limitations. First, it was retrospective in design. Second, some *EGFR* mutant and ICI retreatment patients were enrolled. Therefore, the efficacy might be poor compared with prospective pivotal studies or previous real‐world studies with nivolumab monotherapy. Although the present study revealed that ECOG PS was identified as a significantly favorable prognostic factor for OS and TTF, we were unable to perform multivariate analysis due to the small sample sizes of each ECOG PS subset. Third, some patients were not assessed with regular CT scanning due to clinical practice. Therefore, we could not strictly evaluate progression‐free survival (PFS) and ORR. Finally, a combination regimen of ICI plus platinum doublet chemotherapy has become a standard therapy for first‐line treatment. In patients treated with combination therapy, it might be difficult to apply to our study. However, pembrolizumab monotherapy is still one of the standard regimens for patients with a high expression of PD‐L1 (PD‐L1 ≥ 50%). We expect that our findings could contribute to cases previously treated with pembrolizumab monotherapy as a first‐line treatment.

In conclusion, in previously treated advanced NSCLC patients, atezolizumab monotherapy demonstrated good efficacy and safety regardless of heavily treated patients in real‐world clinical practice, and ECOG PS 0 was a favorable predictive factor. The efficacy of retreatment with atezolizumab was limited but well tolerated in patients treated with prior anti‐PD‐1 antibody.

## CONFLICT OF INTEREST

N. Furuya has received speaker fees as honoraria from AstraZeneca, Chugai, Boehringer Ingelheim Japan, Eli Lilly Japan, Bristol Myers Squibb, Novartis, Taiho, Kyowa Kirin, MSD, and Pfizer.

## References

[1] Herbst RS , Soria JC , Kowanetz M , Fine GD , Hamid O , Gordon MS , et al. Predictive correlates of response to the anti‐PD‐L1 antibody MPDL3280A in cancer patients. Nature. 2014;515(7528):563–7. 25428504 10.1038/nature14011 PMC4836193

[2] Rittmeyer A , Barlesi F , Waterkamp D , Park K , Ciardiello F , von Pawel J , et al. Atezolizumab versus docetaxel in patients with previously treated non‐small‐cell lung cancer (OAK): A phase 3, open‐label, multicentre randomised controlled trial. Lancet. 2017;389(10066):255–65. 27979383 10.1016/S0140-6736(16)32517-X PMC6886121

[3] Mok TSK , Wu YL , Kudaba I , Kowalski DM , Cho BC , Turna HZ , et al. Pembrolizumab versus chemotherapy for previously untreated, PD‐L1‐expressing, locally advanced or metastatic non‐small‐cell lung cancer (KEYNOTE‐042): A randomised, open‐label, controlled, phase 3 trial. Lancet. 2019;393(10183):1819–30. 30955977 10.1016/S0140-6736(18)32409-7

[4] Gandhi L , Rodríguez‐Abreu D , Gadgeel S , Esteban E , Felip E , de Angelis F , et al. Pembrolizumab plus chemotherapy in metastatic non‐small‐cell lung cancer. N Engl J Med. 2018;378(22):2078–92. 29658856 10.1056/NEJMoa1801005

[5] Socinski MA , Jotte RM , Cappuzzo F , Orlandi F , Stroyakovskiy D , Nogami N , et al. Atezolizumab for first‐line treatment of metastatic nonsquamous NSCLC. N Engl J Med. 2018;378(24):2288–301. 29863955 10.1056/NEJMoa1716948

[6] West H , McCleod M , Hussein M , Morabito A , Rittmeyer A , Conter HJ , et al. Atezolizumab in combination with carboplatin plus nab‐paclitaxel chemotherapy compared with chemotherapy alone as first‐line treatment for metastatic non‐squamous non‐small‐cell lung cancer (IMpower130): A multicentre, randomised, open‐label, phase 3 trial. Lancet Oncol. 2019;20(7):924–37. 31122901 10.1016/S1470-2045(19)30167-6

[7] Paz‐Ares L , Luft A , Vicente D , Tafreshi A , Gümüş M , Mazières J , et al. Pembrolizumab plus chemotherapy for squamous non‐small‐cell lung cancer. N Engl J Med. 2018;379(21):2040–51. 30280635 10.1056/NEJMoa1810865

[8] Fujimoto D , Yoshioka H , Kataoka Y , Morimoto T , Kim YH , Tomii K , et al. Efficacy and safety of nivolumab in previously treated patients with non‐small cell lung cancer: A multicenter retrospective cohort study. Lung Cancer. 2018;119:14–20. 29656747 10.1016/j.lungcan.2018.02.017

[9] Kobayashi K , Nakachi I , Naoki K , Satomi R , Nakamura M , Inoue T , et al. Real‐world efficacy and safety of nivolumab for advanced non‐small‐cell lung cancer: A retrospective multicenter analysis. Clin Lung Cancer. 2018;19(3):e349–58. 29398578 10.1016/j.cllc.2018.01.001

[10] Morita R , Okishio K , Shimizu J , Saito H , Sakai H , Kim YH , et al. Real‐world effectiveness and safety of nivolumab in patients with non‐small cell lung cancer: A multicenter retrospective observational study in Japan. Lung Cancer. 2020;40:8–18. 10.1016/j.lungcan.2019.11.014 31838169

[11] Kato R , Hayashi H , Chiba Y , Miyawaki E , Shimizu J , Ozaki T , et al. Propensity score‐weighted analysis of chemotherapy after PD‐1 inhibitors versus chemotherapy alone in patients with non‐small cell lung cancer (WJOG10217L). J Immunother Cancer. 2020;8(1):e000350. 32066647 10.1136/jitc-2019-000350 PMC7057433

[12] Ikeda S , Kato T , Kenmotsu H , Ogura T , Iwasawa S , Sato Y , et al. A phase 2 study of atezolizumab for pretreated NSCLC with idiopathic interstitial pneumonitis. J Thorac Oncol. 2020;15(12):1935–42. 32858235 10.1016/j.jtho.2020.08.018 PMC7446731

[13] Kitagawa S , Hakozaki T , Kitadai R , Hosomi Y . Switching administration of anti‐PD‐1 and anti‐PD‐L1 antibodies as immune checkpoint inhibitor rechallenge in individuals with advanced non‐small cell lung cancer: Case series and literature review. Thorac Cancer. 2020;11(7):1927–33. 32421224 10.1111/1759-7714.13483 PMC7327670

